# Deep Brain Stimulation for Essential Vocal Tremor: A Technical Report

**DOI:** 10.7759/cureus.256

**Published:** 2015-03-10

**Authors:** Allen L Ho, Omar Choudhri, C. Kwang Sung, Elizabeth E DiRenzo, Casey H Halpern

**Affiliations:** 1 Department of Neurosurgery, Stanford University School of Medicine; 2 Neurosurgery, Stanford Medical Center; 3 Department of Otolaryngology - Head & Neck Surgery, Stanford University Medical Center; 4 Department of Neurosurgery, Stanford University Medical Center

**Keywords:** essential vocal tremor, deep brain stimulation, voice, tremor, ventral intermediate nucleus, laryngoscopy, acoustic analysis, awake dbs, microelectrode recording, voice analysis

## Abstract

Essential vocal tremor (EVT) is the presence of a tremulous voice that is commonly associated with essential tremor. Patients with EVT often report a necessary increase in vocal effort that significantly worsens with stress and anxiety and can significantly impact quality of life despite optimal medical and behavioral treatment options. Deep brain stimulation (DBS) has been proposed as an effective therapy for vocal tremor, but very few studies exist in the literature that comprehensively evaluate the efficacy of DBS for specifically addressing EVT. We present a technical report on our multidisciplinary, comprehensive operative methodology for treatment of EVT with frameless, awake deep brain stimulation (DBS).

## Introduction

Tremulous voice is a characteristic feature of several different movement disorders, including essential tremor and Parkinson’s disease, as well as other neurological diseases, such as stroke. One of the clearest associations of tremulous voice occurs in essential tremor and has been referred to as essential vocal tremor (EVT) [1]. Up to 40% of individuals diagnosed with essential tremor also present with EVT [2]. Patients with EVT often report a necessary increase in vocal effort that significantly worsens with stress and anxiety and causes significant social embarrassment. In severe cases, EVT may result in discontinuation of employment and hobbies, and thus has a significant impact on quality of life [3].

In EVT, alterations in the pitch and/or intensity of the voice are caused by rhythmic oscillations at a rate of 4-8 Hz of the laryngeal, pharyngeal and/or palatal muscles. These changes correlate with acoustic modulations of fundamental frequency (*f*_0_) for pitch and amplitude for loudness. These vocal symptoms are typically most prominent with the sustained phonation of vowels, though they are present to some degree across all phonatory activities [1, 3]. Typical oral medication-based treatments for essential tremor have not been shown to be effective at addressing vocal tremor. Botulinum toxin injected into the thyroarytenoid and extralaryngeal muscles is commonly employed for treatment of EVT and has been shown to be effective in 56-80% of patients [4]. This treatment results in a decrease in tremor amplitude; however, effects are transient, necessitating repeat injections, and a complete resolution of vocal instability is not achieved. Furthermore, these changes do not always translate into acoustic improvement or greater voice functionality. Side-effects, such as prolonged breathiness, coughing, choking and dysphagia, are the main limiting factor with botulinum injections, especially in the elderly population. [5] Thus, there clearly exists a need for a more effective, safe and permanent solution for EVT.

We present a technical report on our multidisciplinary, comprehensive operative methodology for treatment of EVT with frameless, awake deep brain stimulation (DBS).

## Technical report

### Patient evaluation

Patient eligibility for DBS for EVT will be determined by a comprehensive, multi-disciplinary outpatient evaluation. Patients will have progressive, debilitating vocal tremor (and in some cases, other types of tremor, such as extremity and head tremors) that significantly interferes with daily living, and is refractory to optimal medical management with medications, such as propranolol and/or primidone [6]. In addition to neurosurgical evaluation, patients will have to be seen by both a laryngologist and a speech language pathologist for a full vocal tremor work-up. The laryngologist will perform a preoperative flexible distal-chip laryngoscopy to detail the anatomic etiology of the vocal tremor. Laryngoscopy will reveal characteristic rhythmic, oscillatory motion of the palate, pharynx, or vocal folds during a sustained phonatory vowel task [3]. The preoperative speech language pathology work-up will describe the exact acoustic and instrumental voice related characteristics of the vocal tremor in an effort to provide objective, clinically significant data about changes in voice with DBS both intra- and postoperatively.

### DBS placement

The methodology and technique for awake frameless DBS has been extensively described previously [7-8], but we will detail our methodology with a specific focus on ventral intermediate nucleus (VIM) DBS targeting for vocal tremor. The location of the VIM nucleus varies by individual, but is targeted approximately 12 mm lateral to the anterior commissure - posterior commissure (AC-PC) line and 6 mm posterior to the mid-point of the AC-PC [9]. Given the medial-to-lateral facial-forelimb-hindlimb somatotopy of the thalamic motor nuclei, targets for both stimulator placements are chosen approximately 1-2 mm medial to the typical target for essential tremor in order to suppress voice tremor [10-11].

The surgery must be done in the awake state so as to ensure adequate control of vocal tremor by a speech pathologist. The large majority of patients have concomitant head and limb tremor. Thus, the awake patient is required to ensure adequate control of all or most areas of tremor, including vocal tremor. The depth of the planned medial target within VIM is calculated and entered into the microdrive with an initial depth of the microelectrode set to 15 mm above target. The microelectrode is then tested and its impedance range confirmed after conditioning. The microelectrode is then advanced in a stepwise fashion, continuously recording. Excellent single unit recordings are obtained as the electrode is advanced through the thalamus. With the aid of a neurophysiologist, kinesthetic responses encountered for the expected corresponding extremity and more proximal motor groups, neck, and face with passive range of motion, as well as with macrostimulation through the microcannula, are recorded. Macrostimulation with careful electrophysiologic documentation of paresthesias and tremor reduction/resolution is conducted as the electrode approaches the planned target trajectory. With respect to voice tremor, a speech evaluation is performed with a full intraoperative acoustic evaluation done by a speech language pathologist.

For evaluation of EVT, rate and magnitude of the *f*_0_ and amplitude modulations, which tend to co-occur, are measured and recorded [1, 12]. These measurements are the most common acoustic characteristics of vocal tremor and improvement from baseline preoperative measurements confirm adequate electrode and lead placement to specifically address vocal tremor. Additional instrumental measures of voice acoustics, including jitter, shimmer, and harmonic-to-noise ratio, and voice aerodynamics, such as maximum phonation time and s/z ratio, are also gathered to confirm improvement in both instrumental and aerodynamic measures of vocal tremor from preoperative evaluation. Elevated jitter and shimmer and decreased harmonic-to-noise ratio, s/z ratio, and maximum phonation time are typical instrumental voice findings of patients with EVT [13].

After microrecording and macrostimulation confirms adequate placement of the electrode with good vocal tremor response, the lead may be implanted along this tract. Multiple passes, while not without additional risk of morbidity [14], may be required to achieve optimal electrode placement for vocal tremor control. After appropriate attenuation of voice tremor and/or any other anatomical tremor, a Medtronic 3389 DBS stimulation electrode is measured to the appropriate length and introduced into the target point of this track. Test stimulations are done to rule out adverse effects and confirm therapeutic benefit to extremity, head, and vocal tremors. The lead position is then confirmed and secured in place.  The same procedure is repeated for the opposite side for those patients with bilateral tremor. It remains unclear if vocal tremor responds to unilateral DBS.

The patient is then brought back at a one-week interval, for infraclavicular pulse generator implantation, which is performed in standard fashion [15]. Programming of the bilateral VIM DBS are performed two weeks from implantation with both neurophysiologic and comprehensive speech analysis (see below) to confirm adequate control of vocal tremor.

## Discussion

EVT is a difficult-to-treat voice disorder that significantly interferes with the quality of life of many afflicted patients despite optimal medical and behavioral treatment options [1]. DBS has been proposed as an effective therapy for vocal tremor, but very few studies exist in the literature that comprehensively evaluate the efficacy of DBS for specifically addressing EVT. Sataloff, et. al. first published on two cases of DBS specifically for the treatment of vocal tremor in 2002, in which two patients underwent bilateral stimulator implantation in the ventral intermediate nucleus (VIM) and were evaluated by strobovideolaryngoscopy and objective voice analysis. Vocal tremor was eliminated completely in one patient and significantly decreased in the other [16]. Most other published data on reduction of voice tremor following DBS have been for patients with varied pathology, and are typically not comprehensive evaluations with nasal endoscopy for direct laryngeal visualization, as well as instrumental voice assessments, including acoustics and aerodynamics [17-21]. We present a technical report detailing the methodology for comprehensive assessment of EVT prior to, during, and following frameless, awake DBS surgery. This methodology is the basis of our multidisciplinary, comprehensive DBS program for the treatment of ETV, the results of which will assist in the development of evidence-based guidelines regarding new DBS treatment paradigms for these difficult-to-serve patients.

## Conclusions

Though DBS has been utilized in the treatment of vocal tremor associated with various movement disorders, very few studies specifically examine the efficacy of this treatment with comprehensive laryngoscopic and objective voice analysis. We present a technical report of our multidisciplinary, comprehensive operative methodology for treatment of EVT with frameless, awake DBS paired with a comprehensive laryngoscopic and vocal acoustic analysis to identify the optimal intraoperative stimulation targets and objectively quantify improvements in voice control. Given the encouraging results from our initial experience with this methodology, a continued prospective study of DBS for EVT is currently underway at our institution to more robustly quantify the efficacy of this treatment modality.
